# Effect on Osteogenic Differentiation of Genetically Modified IL4 or PDGF-BB Over-Expressing and IL4-PDGF-BB Co-Over-Expressing Bone Marrow-Derived Mesenchymal Stromal Cells In Vitro

**DOI:** 10.3390/bioengineering8110165

**Published:** 2021-10-29

**Authors:** Masanori Tsubosaka, Masahiro Maruyama, Elijah Ejun Huang, Ning Zhang, Takeshi Utsunomiya, Qi Gao, Huaishuang Shen, Xueping Li, Junichi Kushioka, Hirohito Hirata, Zhenyu Yao, Yunzhi Peter Yang, Stuart B. Goodman

**Affiliations:** 1 Department of Orthopaedic Surgery, Stanford University School of Medicine, 450 Broadway Street, Redwood City, CA 94063, USA; masanor1@stanford.edu (M.T.); masa.maruyama1460@gmail.com (M.M.); eehuang@stanford.edu (E.E.H.); ningzzz@stanford.edu (N.Z.); takeshiu0625@gmail.com (T.U.); qigao7@stanford.edu (Q.G.); hss2018@stanford.edu (H.S.); xuepli@stanford.edu (X.L.); junichi5@stanford.edu (J.K.); hirohith@stanford.edu (H.H.); zhenyuy@stanford.edu (Z.Y.); ypyang@stanford.edu (Y.P.Y.); 2 Department of Material Science and Engineering, Stanford University School of Medicine, Stanford, CA 94305, USA; 3 Department of Bioengineering, Stanford University School of Medicine, Stanford, CA 94305, USA

**Keywords:** mesenchymal stromal cells, genetically modified, preconditioning, osteonecrosis, core decompression

## Abstract

The use of genetically modified (GM) mesenchymal stromal cells (MSCs) and preconditioned MSCs (pMSCs) may provide further opportunities to improve the outcome of core decompression (CD) for the treatment of early-stage osteonecrosis of the femoral head (ONFH). GM interleukin-4 (IL4) over-expressing MSCs (IL4-MSCs), platelet-derived growth factor (PDGF)-BB over-expressing MSCs (PDGF-BB-MSCs), and IL4-PDGF-BB co-over-expressing MSCs (IL4-PDGF-BB-MSCs) and their respective pMSCs were used in this in vitro study and compared with respect to cell proliferation and osteogenic differentiation. IL4-MSCs, PDGF-BB-MSCs, IL4-PDGF-BB-MSCs, and each pMSC treatment significantly increased cell proliferation compared to the MSC group alone. The percentage of Alizarin red-stained area in the IL4-MSC and IL4-pMSC groups was significantly lower than in the MSC group. However, the percentage of Alizarin red-stained area in the PDGF-BB-MSC group was significantly higher than in the MSC and PDGF-BB-pMSC groups. The percentage of Alizarin red-stained area in the IL4-PDGF-BB-pMSC was significantly higher than in the IL4-PDGF-BB-MSC group. There were no significant differences in the percentage of Alizarin red-stained area between the MSC and IL4-PDGF-BB-pMSC groups. The use of PDGF-BB-MSCs or IL4-PDGF-BB-pMSCs increased cell proliferation. Furthermore, PDGF-BB-MSCs promoted osteogenic differentiation. The addition of GM MSCs may provide a useful supplementary cell-based therapy to CD for treatment of ONFH.

## 1. Introduction

Osteonecrosis of the femoral head (ONFH) is a debilitating disease in which the femoral head may collapse, subsequently causing osteoarthritis of the hip [1]. This disease has important consequences for healthcare delivery and major social and financial implications for approximately 20 million ONFH patients worldwide [2,3]. Early diagnosis of ONFH is essential to prevent progressive disease, which causes a structural collapse of the femoral head and subchondral fracture [4]. Core decompression (CD) is a joint-sparing surgery performed in the early stages of ONFH to help arrest the progression of disease [1]. The success rate of CD for ONFH reaches about 70% at a follow-up of about five years, after which additional surgery is often not necessary [5]. To increase the success rate of CD for ONFH, it is essential to validate synergistic, evidence-based methods, such as cell-based therapies, that provide further biological augmentation to CD in order to optimize bone regeneration [6].

The role of mesenchymal stromal cells (MSCs) in the pathogenesis of osteonecrosis has only recently received attention; it has been reported that ONFH inhibits the differentiation of osteoblasts from mesenchymal progenitor cells [7]. The capillaries, which act as conduits for delivering stem cells and osteocytes for the bone repair unit, may also be occluded by thrombi or emboli in ONFH [8]. Methods to regulate biological, biochemical, and biophysical factors to influence MSC function and lineage-specific differentiation to increase their therapeutic potential have been reported [9]. One example is preconditioning with inflammatory mediators. This preconditioning method is widely used in immunology and has also been applied to various stem cells [10,11]. We developed a novel preconditioning strategy using tumor necrosis factor-alpha (TNFα) and lipopolysaccharide (LPS) to enhance both the regenerative and immunomodulatory abilities of MSCs to form bone. We reported that MSC-based therapies using the above preconditioning method might be applicable to treating inflammatory diseases such as ONFH [12].

The MSC secretome, which contains a variety of molecules such as cytokines and growth factors, has attracted much attention in recent years because the secretome has been reported to improve tissue repair [13]. The MSC secretome is contained in the conditioned medium during cell culture. This conditioned medium has been proven to be useful for bone regeneration [14]. The bone regeneration capacity of various scaffolds enriched with a conditioned medium has been demonstrated to enhance osteogenesis [15].

Based on the interaction of MSCs with cells of the immune system, regeneration occurs in damaged mesenchymal tissues, including bone. MSCs and other progenitors are ultimately involved in the regeneration of bone and other tissues, and macrophages have been shown to play an essential role in modulating the mobilization and differentiation of these cells. MSCs and macrophages coordinate their functions with each other to facilitate the physiological transition from acute inflammation to tissue regeneration [16]. We considered the use of anti-inflammatory cytokines such as interleukin-4 (IL4) as an essential strategy to resolve inflammation for chronic inflammatory bone diseases and related conditions. In vivo studies in a mouse model of chronic inflammation caused by wear-particles showed that IL4 promotes bone formation and prevents bone loss by changing the polarity of local M0 or M1 macrophages to an M2 phenotype [17]. Therefore, to further enhance the immunomodulatory function of MSCs, we established IL-4 over-expressing lentiviral vectors and introduced them into the MSCs [18]. Genetically modified IL4 over-expressing MSCs (IL4-MSC) showed stronger immunomodulation and promoted early bone formation in response to inflammation caused by titanium particles [19].

Based on these findings, we investigated the efficacy of CD in combination with MSCs, preconditioned MSCs (pMSCs), and IL4-MSCs with injectable hydrogels as a cell-based therapy for the treatment of steroid-associated ONFH in rabbits. Although the injection of pMSCs into the bone tunnel significantly enhanced osteogenesis of the femoral head in vivo compared to MSCs, there was no significant difference in the number of empty lacunae, a histological marker of bone cell death. The injection of IL4-MSCs significantly decreased the number of empty lacunae compared to MSCs but resulted in less osteogenesis [20].

Platelet-derived growth factor (PDGF)-BB promotes both angiogenesis and osteogenesis [21]. PDGF-BB also improves bone formation and bone strength in mice [22]. In addition, PDGF-BB is thought to have beneficial immunomodulatory effects. PDGF-BB inhibits interleukin-1β (IL-1β)-induced NF-κB activation and apoptosis, and the detrimental impact of IL-1β on chondrocytes is mitigated by PDGF-BB supplementation, indicating that PDGF-BB contributes to an anti-inflammatory microenvironment [23]. We also demonstrated that genetically modified PDGF-BB over-expressing MSCs (PDGF-BB-MSCs) promoted increased osteogenesis, and IL4 and PDGF-BB co-over-expressing MSCs (IL4-PDGF-BB-MSCs) had the potential to mitigate the inhibitory effect of IL4 on osteogenesis by IL4 over-expressing MSCs in vitro [24].

This in vitro study investigated the efficacy of genetically modified over-expressing MSCs, i.e., IL4-MSCs, PDGF-BB-MSCs, and IL4-PDGF-BB-MSCs and pMSCs for use as adjunctive cell-based therapy for CD.

## 2. Materials and Methods

### 2.1. Rabbit Bone Marrow-Derived MSCs

We used rabbit bone marrow-derived MSCs purchased from Cyagen Biosciences (Santa Clara, CA, USA) in all experiments. MSCs were expanded with MSC growth medium (α-Minimal Essential Medium—α-MEM, Thermo Fisher Scientific, Rockford, IL, USA; supplemented 10% MSC certified bovine serum—FBS, Life Technologies, Pleasanton, CA, USA; 1% antibiotic and antimycotic solution—100 units of penicillin, 100 μg of streptomycin, and 0.25 of amphotericin B per milliliter, Life Technologies) until passage 5.

### 2.2. Construction of Plasmids

The vectors containing elements of rabbit IL-4 (rIL-4) or human PDGF-BB (hPDGF-BB) were generated according to our previous report [24]. Briefly, the rIL-4 fragment was amplified by polymerase chain reaction (PCR) from a rabbit IL-4 cDNA ORF clone (OOb30690, GenScript, Piscataway, NJ, USA) and inserted into a pCDH-CMV-MCS-copGFP lentiviral expression vector with a cytomegalovirus (CMV) promoter (CD511B-1; System Biosciences, Palo Alto, CA, USA) to generate the pCDH-CMV-rIL4-copGFP vector. A fragment containing the hPDGF-BB element was amplified by PCR from the plasmid pBabe-PDGF/B-zeo (Plasmid #17757, Addgene, Watertown, MA, USA) and inserted into the pCDH-CMV-MCS-copGFP or pCDH-CMV-MCS-mRFP lentiviral expression vectors to generate the pCDH-CMV-hPDGF-BB-copGFP or pCDH-CMV-hPDGF-BB-mRFP vectors, respectively. The CMV promoter in the pCDH-CMV-hPDGF-BB-copGFP and pCDH-CMV-hPDGF-BB-mRFP lentiviral expression vectors was replaced with a fragment containing a weak phosphoglycerate kinase (PGK) obtained from pCDH-PGK (Plasmid #72268, Addgene, Watertown) to generate pCDH-PGK-hPDGF-BB-copGFP and pCDH-PGK-hPDGF-BB-mRFP lentiviral expression vectors, respectively. These vectors were amplified and obtained by competent Escherichia coli bacterial cells (One Shot™ Stbl3™ Chemically Competent E. coli; Invitrogen, Thermo Fisher Scientific, Waltham, MA, USA).

### 2.3. Establishment of Genetically Modified Control MSCs (GFP-MSCs), IL4-MSCs, and PDGF-BB-MSCs

The lentivirus vector pCDH-CMV-copGFP for control, the lentiviral vector pCDH-CMV-rIL4-copGFP for rabbit IL4 secretion, or the lentiviral vector pCDH-PGK-hPDGF-BB-copGFP for human PDGF-BB secretion were produced in human embryonic kidney 293 T cells (ATCC, Manassas, VA, USA) by co-transfecting with the control, rabbit IL4 over-expressing, or human PDGF-BB over-expressing transfer plasmid, packaged plasmid (psPAX2), and enveloped plasmid (pMD2G VSVG) using a calcium phosphate transfection kit (Takara Bio USA, Inc., Mountain View, CA, USA) with 25 mmol/L chloroquine. The virus was mixed in a serum-free medium with 6 μg/mL of polybrene (Sigma Aldrich, St. Louis, MO, USA) and incubated at the multiplicity of infection (MOI) of 80 for 6 h to infect into rabbit MSCs. The infected cells were confirmed to be GFP-positive cells by fluorescence microscope on day 4 post-infection. These established MSCs were used from passages 8–10. IL4-MSCs or PDGF-BB-MSCs were also used after testing the IL4 or PDGF-BB secreting level with enzyme-linked immunosorbent assay (ELISA).

### 2.4. Establishment of IL4-PDGF-BB-MSCs

**Figure 1:**
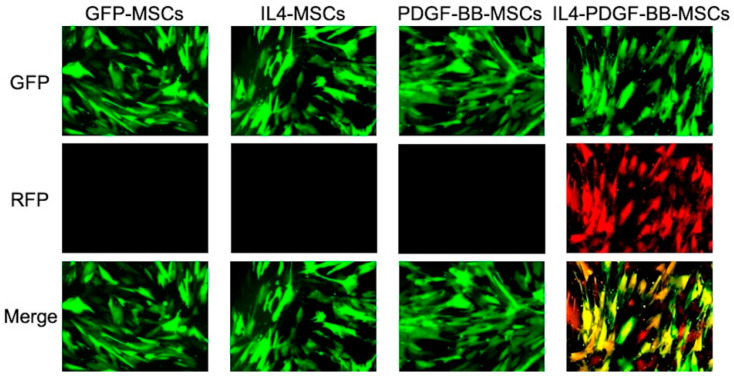
Representative fluorescence microscopy images of mesenchymal stromal cells (MSCs) infected by IL-4 and/or PDGF-BB lentiviral vectors. GFP-MSCs, MSCs infected with control GFP-positive lentivirus vector; IL4-MSCs, MSCs infected with rIL-4 secreting GFP-positive lentivirus vector; PDGF-BB-MSCs, MSCs infected with hPDGF-BB secreting GFP-positive lentivirus vector; IL4-PDGF-BB-MSCs, previously established IL4-MSCs infected with hPDGF-BB secreting RFP-positive lentivirus vector.

The lentiviral vector pCDH-PGK-hPDGF-BB-mRFP for human PDGF-BB secretion was produced in human embryonic kidney 293 T cells (ATCC, Manassas) by co-transfecting with the human PDGF-BB over-expressing transfer plasmid, packaged plasmid (psPAX2), and enveloped plasmid (pMD2G VSVG) using a calcium phosphate transfection kit (Takara Bio USA, Inc., Mountain View, CA, USA) with 25 mmol/L chloroquine. The virus was mixed in a serum-free medium with 6 μg/mL of polybrene (Sigma Aldrich, St. Louis, MO, USA) and incubated at the MOI of 80 for 6 h to infect into previously established rabbit IL4-MSCs. The infected cells were confirmed to be GFP- or RFP-positive cells by fluorescence microscope on day 4 post-infection. IL4-PDGF-BB-MSCs from passages 8–10 were used after testing the IL4 and PDGF-BB secreting levels with ELISA. Thus, four kinds of MSCs were established in this study (Figure 1).

### 2.5. Cell Preparation for In Vitro Experiments

GFP-MSCs, IL4-MSCs, PDGF-BB-MSCs, or IL4-PDGF-BB-MSCs were seeded into T75 flasks and cultured in an MSC growth medium for 1 day.

In the GFP-MSC, IL4-MSC, PDGF-BB-MSC, and IL4-PDGF-BB-MSC groups, the medium was replaced with a fresh MSC growth medium, and the cells were cultured for 3 more days. The cells from these four groups were washed three times with Dulbecco’s phosphate-buffered saline (DPBS), then the cells were trypsinized and seeded for each experiment.

**Figure 2:**
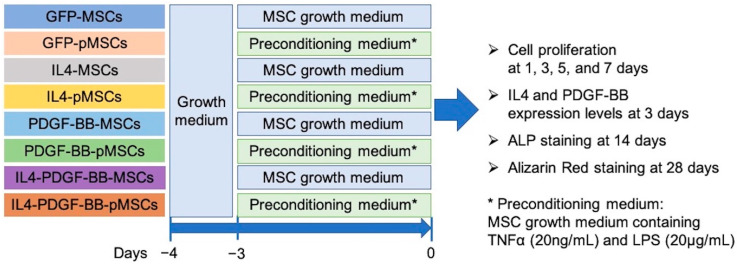
The experimental outline of in vitro experiments. GFP-MSCs, IL4-MSCs, PDGF-BB-MSCs, or IL4-PDGF-BB-MSCs were seeded into T75 flasks and cultured in an MSC growth medium for 1 day. For the non-preconditioning MSC groups, the cells were cultured for 3 days with a fresh MSC growth medium. For the preconditioning MSC groups, cells were cultured in the MSC preconditioning medium for 3 days. The cells from all eight groups were washed three times with Dulbecco’s phosphate-buffered saline. The cells were trypsinized and seeded for each experiment, including cell proliferation, IL4 and PDGF-BB expression level, and osteogenic differentiation performed by alkaline phosphatase (ALP) staining and Alizarin red-staining. Abbreviations: MSCs, mesenchymal stromal cells; pMSCs, preconditioning MSCs; TNFα, tumor necrosis factor-alpha; LPS, lipopolysaccharide.

Preconditioning must always be performed immediately before use. To precondition the GFP-MSC, IL4-MSC, PDGF-BB-MSC, and IL4-PDGF-BB-MSC groups, the cells 1 day after seeding were cultured in the MSC pre-conditioning medium (MSC growth medium containing TNFα (20 ng/mL) and LPS (20 μg/mL)) for 3 days. The resulting GFP-pMSCs, IL4-pMSCs, PDGF-BB-pMSCs, and IL4-PDGF-BB-pMSCs were washed three times with DPBS, then the cells were trypsinized and seeded for each experiment. Figure 2 shows the experimental outline of in vitro experiments.

### 2.6. Cell Proliferation

The cells from all eight groups (GFP-MSCs, GFP-pMSCs, IL4-MSCs, IL4-MSCs, PDGF-BB-MSCs, PDGF-BB-pMSCs, IL4-PDGF-BB-MSCs, and IL4-PDGF-BB-MSC) were washed three times with DPBS, and then the cells were trypsinized and seeded in a 6-well plate (2 × 10^4^ cells/well) using the MSC growth medium. The cells were used on days 1, 3, 5, and 7. For the day 7 cells, the medium was replaced with fresh medium on day 3. Cell proliferation was analyzed using the Quant-iT PicoGreen dsDNA Assay Kit (Invitrogen, Carlsbad, CA, USA). After washing the cells seeded in a 6-well plate three times with DPBS, 1 mL of DNase/RNase-free water was added to each well. The freeze/thaw cycle was repeated three times to release cellular DNA, which was stained with PicoGreen according to the manufacturer’s protocol. The amount of dsDNA was calculated by measuring the fluorescence at the wavelength of 480/520 nm using a plate reader (SpectraMax M2e Microplate Reader; Molecular Devices, San Jose, CA, USA).

### 2.7. IL4 and PDGF-BB Expression Levels

The cells from all eight groups (GFP-MSCs, GFP-pMSCs, IL4-MSCs, IL4-MSCs, PDGF-BB-MSCs, PDGF-BB-pMSCs, IL4-PDGF-BB-MSCs, and IL4-PDGF-BB-MSC) were washed three times with DPBS, and then the cells were trypsinized and seeded in a 6-well plate (2 × 10^4^ cells/well) using the MSC growth medium. The supernatants on day 3 of the IL4-MSC, IL4-pMSC, PDGF-BB-MSC, PDGF-BB-pMSC, IL4-PDGF-BB-MSC, and IL4-PDGF-BB-pMSC groups were used. The expression levels of IL4 and PDGF-BB on day 3 were measured using the rabbit IL4 ELISA Kit (R&D Systems, Minneapolis, MN, USA) and the human PDGF-BB ELISA kit (R&D Systems, Minneapolis), respectively, according to the manufacturer’s protocols. The optical absorbance at 450 nm was measured using a plate reader (SpectraMax M2e Microplate Reader; Molecular Devices). The IL4 expression level-dsDNA ratio or PDGF-BB expression level-dsDNA ratio were also calculated using the absolute value of IL4 or PDGF-BB expressions divided by the amount of dsDNA, respectively.

### 2.8. Osteogenic Differentiation

The cells from all eight groups were washed three times with DPBS, and then the cells were trypsinized and seeded in 24-well plates with 4 × 10^4^ cells/well for alkaline phosphatase (ALP) staining and with 2 × 10^4^ cells/well for Alizarin red-staining. Cells were cultured in the osteogenic medium (α-MEM supplemented with 10% MSC certified FBS, 1% antibiotic and antimycotic solution, 50 mM l-ascorbic acid, Sigma-Aldrich; 100 mM Vitamin D3, Sigma-Aldrich; 100 nM dexamethasone, Sigma-Aldrich; 10 mM β-glycerophosphate, MP Biomedicals). The medium was changed to a fresh medium twice a week.

ALP staining was performed 2 weeks after seeding. Cells were fixed in 4% paraformaldehyde (PFA) for 30 min, washed three times with DPBS, and stained with ALP substrate solution (1-StepTM NBT/BICP substrate Solution, Thermo Fisher Scientific, Waltham, MA, USA) at 37 °C overnight. After washing three times with DPBS and drying, photographs of the entire wells were taken using a BZ-X 810 digital microscope (Keyence, Osaka, Japan). Nine 20x micrographs were taken and then stitched together to create each photograph of the entire well. The percentage of ALP-stained area per well was analyzed using ImageJ 1.53e (NIH).

Alizarin red-staining was performed 4 weeks after seeding. Cells were fixed in 2% PFA for 10 min, washed three times with DPBS, and stained with 40 mM pH 4.1–4.3 Alizarin Red S (Sigma-Aldrich, St. Louis, MO, USA) for 15 min. After washing three times with DPBS, photographs of the entire wells were taken using a BZ-X 810 digital microscope (Keyence, Osaka, Japan). Nine 20x micrographs were taken and then stitched together to create each photograph of the entire well. The percentage of Alizarin red-stained area per well was analyzed using ImageJ 1.53e (NIH).

### 2.9. Statistical Analysis

All data are shown as mean ± standard error. All the in vitro experiments were performed in triplicate. A one-way ANOVA with post-hoc Tukey test was performed with IBM SPSS statistical software (version 21; IBM Corp., Armonk, NY, USA). Statistical significance was set at *p* < 0.05.

## 3. Results

### 3.1. Cell Proliferation

**Figure 3:**
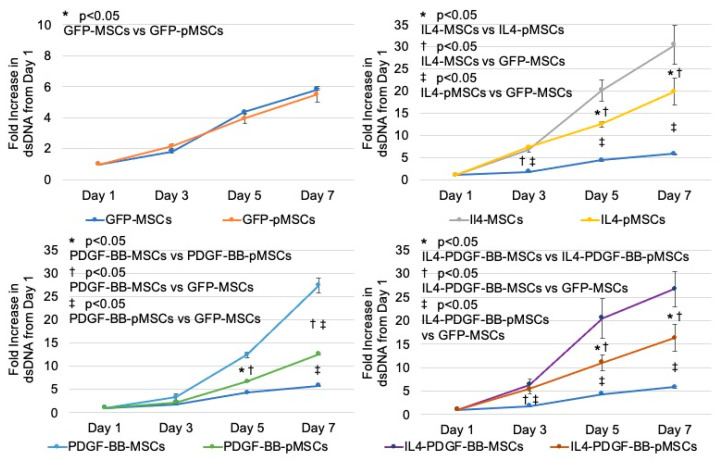
Cell proliferation measured by PicoGreen dsDNA quantitation assay.

The fold changes in the amount of dsDNA on days 3, 5, and 7 compared to day 1 are shown in Table 1. IL4-MSC, PDGF-BB-MSC, and IL4-PDGF-BB-MSC groups resulted in significantly accelerated cell proliferation compared to each pMSC group on days 5 and 7. The three genetically modified pMSCs also significantly accelerated cell proliferation compared to the GFP-MSC group on day 7 (Figure 3).

### 3.2. IL4 and PDGF-BB Expression Levels

**Figure 4:**
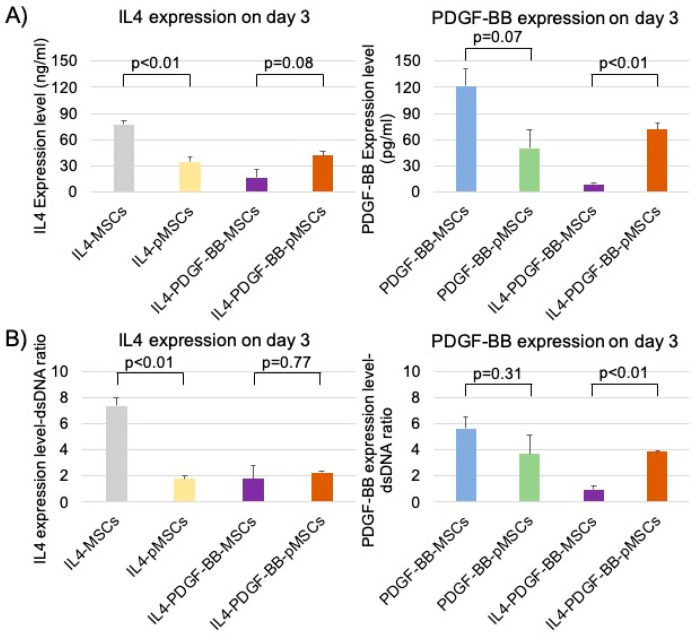
(**A**) IL4 and PDGF-BB expression levels on day 3 in the IL4-MSC, IL4-pMSC, PDGF-BB-MSC, PDGF-BB-pMSC, IL4-PDGF-BB-MSC, and IL4-PDGF-BB-pMSC groups. IL4 expression levels on day 3 in the IL4-MSC, IL4-pMSC, IL4-PDGF-BB-MSC, and IL4-PDGF-BB-pMSC groups were 77.7 ± 4.4 ng/mL, 34.6 ± 5.7 ng/mL, 16.8 ± 9.4 ng/mL, and 41.8 ± 5.0 ng/mL, respectively. PDGF-BB expression levels on day 3 in the PDGF-BB-MSC, PDGF-BB-pMSC, IL4-PDGF-BB-MSC, and IL4-PDGF-BB-pMSC groups were 121.7 ± 20.1 pg/mL, 50.3 ± 20.4 pg/mL, 8.7 ± 2.0 pg/mL, 71.5 ± 7.8 pg/mL, respectively. (**B**) IL4 and PDGF-BB expression level-dsDNA ratios on day 3 in the IL4-MSC, IL4-pMSC, PDGF-BB-MSC, PDGF-BB-pMSC, IL4-PDGF-BB-MSC, and IL4-PDGF-BB-pMSC groups. IL4 expression level-dsDNA ratios on day 3 in the IL4-MSC, IL4-pMSC, IL4-PDGF-BB-MSC, and IL4-PDGF-BB-pMSC groups were 7.3 ± 0.6, 1.8 ± 0.2, 1.8 ± 1.0, and 2.3 ± 0.1, respectively. PDGF-BB expression level-dsDNA ratios on day 3 in the PDGF-BB-MSC, PDGF-BB-pMSC, IL4-PDGF-BB-MSC, and IL4-PDGF-BB-pMSC groups were 5.6 ± 0.9, 3.7 ± 1.4, 1.0 ± 0.3, and 3.9 ± 0.1, respectively.

The IL4 expression level on day 3 in the IL4-MSC group was significantly higher than the IL4-pMSC group. The PDGF-BB expression level on day 3 in the PDGF-BB-MSC group was higher than the PDGF-BB-pMSC group. On the other hand, the IL4 expression level on day 3 in the IL4-PDGF-BB-pMSC group was higher than the IL4-PDGF-BB-MSC group. Furthermore, the PDGF-BB expression level on day 3 in the IL4-PDGF-BB-pMSC group was significantly higher than the IL4-PDGF-BB-MSC group (Figure 4A). 

The same tendency as each absolute value of IL4 and PDGF-BB expression levels was observed in the IL4 and PDGF-BB expression level-dsDNA ratio among IL4-MSC, PDGF-BB-MSC, and IL4-PDGF-BB-MSC groups (Figure 4B).

### 3.3. Osteogenic Differentiation

#### 3.3.1. ALP Staining

**Figure 5:**
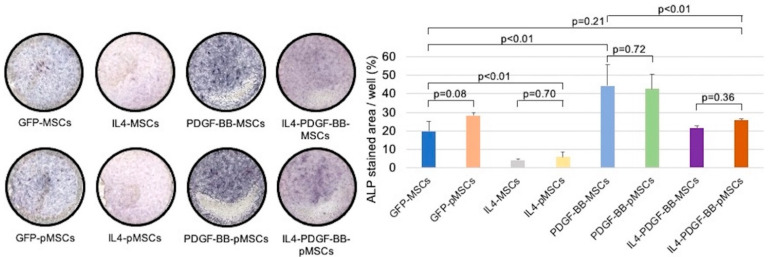
Alkaline phosphatase-staining in all eight groups 2 weeks after seeding. The percentages of stained area in the GFP-MSC, GFP-pMSC, IL4-MSC, IL4-pMSC, PDGF-BB-MSC, PDGF-BB-pMSC, IL4-PDGF-BB-MSC, and IL4-PDGF-BB-pMSC groups were 19.8 ± 5.3%, 28.3 ± 1.4%, 4.3 ± 0.7%, 6.0 ± 2.4%, 44.2 ± 11.7%, 42.5 ± 8.1%, 21.4 ± 1.5%, and 25.7 ± 1.0%, respectively. Abbreviations: ALP, alkaline phosphatase.

The percentage of stained area in the IL4-MSC and IL4-pMSC groups was significantly lower than the GFP-MSC group. However, the percentage of stained area in the PDGF-BB-MSC and PDGF-BB-pMSC groups was significantly higher than the GFP-MSC group. There were no significant differences in the percentage of stained area among the GFP-MSC, IL4-PDGF-BB-MSC, and IL4-PDGF-BB-pMSC groups. The percentage of stained area in the PDGF-BB-MSC group was also significantly higher than the IL4-PDGF-BB-pMSC group (Figure 5).

#### 3.3.2. Alizarin Red Staining

**Figure 6:**
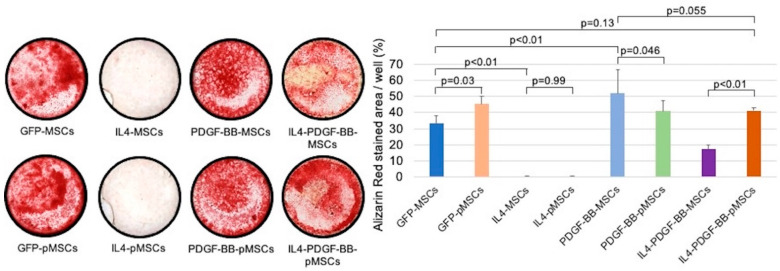
Alizarin red-staining in all eight groups 4 weeks after seeding. The percentages of stained area in the GFP-MSC, GFP-pMSC, IL4-MSC, IL4-pMSC, PDGF-BB-MSC, PDGF-BB-pMSC, IL4-PDGF-BB-MSC, and IL4-PDGF-BB-pMSC groups were 33.1 ± 4.7%, 45.1 ± 4.9%, 0.64 ± 0.08%, 0.58 ± 0.03%, 51.7 ± 14.5%, 40.7 ± 6.7%, 17.3 ± 2.5%, and 41.2 ± 1.7%, respectively.

The percentage of stained area in the IL4-MSC and IL4-pMSC groups was significantly lower than the GFP-MSC group. However, the percentages of stained area in the PDGF-BB-MSC and PDGF-BB-pMSC groups were significantly higher than the GFP-MSC group. The percentage of stained area in the IL4-PDGF-BB-pMSC was significantly higher than in the IL4-PDGF-BB-MSC group. There was no significant difference in the percentage of the stained area between the GFP-MSC and IL4-PDGF-BB-pMSC groups. The percentage of stained area in the PDGF-BB-MSC group was higher than the IL4-PDGF-BB-pMSC with no statistical difference (Figure 6).

The summary of all the above results is shown in Table 2.

## 4. Discussion

Various methods have been developed to increase the survival of transplanted cells and promote tissue regeneration at the transplant site [25]. One example is the preconditioning method of MSCs [26,27], which involves exposing cells to physical and environmental stresses [10] and pharmacological modulation of target molecules [11]. We focused on the method of pharmacological preconditioning of MSCs before transplantation and developed a novel preconditioning strategy using TNFα and LPS to enhance both the bone regenerative and immunomodulatory abilities of MSCs [12]. In our study, there was no difference in cell proliferation between GFP-MSC and GFP-pMSC groups. Due to the pro-inflammatory environment of preconditioning, inflammatory cytokines from macrophages and other white blood cells subsequently activate or “license” MSCs to become anti-inflammatory, immune modulators [28,29]. The pMSCs change their phenotype which encourages their differentiation rather than proliferation, and therefore, the pMSCs might not show any difference in cell proliferation from MSCs. Still, the GFP-pMSC group showed better osteogenic differentiation than the GFP-MSCs group. The proinflammatory cytokine TNF-α has been reported to stimulate osteogenic differentiation of MSCs [30,31]. The proinflammatory agent LPS has also been reported to promote osteogenic differentiation of MSCs through the activation of Toll-Like Receptor 4 [32]. The reason why pMSCs showed better osteogenic differentiation than MSCs in our study might be due to our preconditioning strategy using TNFα and LPS.

The purpose of gene modification of MSCs is to facilitate MSC survival, affect their capacity for differentiation, regulate the expression of specific genes and alter the secretion of specific proteins [33,34,35]. Our previous study showed that genetically modified MSCs can survive in vivo for a long time (over a month) [36]. We have established three types of genetically modified rabbit MSCs in this study, namely, IL4-MSCs, PDGF-BB-MSCs, and IL4-PDGF-BB-MSCs. Regarding cell proliferation, these three MSCs accelerated cell proliferation compared to the GFP-MSC group. IL4 has been reported to promote cell proliferation [37,38], and we have also previously reported that IL4 promotes the proliferative capacity of MSCs [20]. PDGF-BB is a potent growth promoter of MSCs and has been reported to efficiently stimulate cell proliferation [39,40]. The expressions of IL4 and/or PDGF-BB in the three types of MSCs explored in this study stimulated cell proliferation for each specific type of MSC compared to GFP-MSCs. However, these three pMSC types also decreased cell proliferation compared to each corresponding gene modified MSC type without preconditioning on day 7. Previously, we reported that the proliferative ability of IL4-pMSCs was inferior to that of IL4-MSCs [20]. It appears that the cell proliferative ability of genetically modified MSCs may be somewhat reduced when they are preconditioned.

Regarding osteogenic differentiation, the percentage of ALP and Alizarin red-stained area in the IL4-MSC and IL4-pMSC groups was lower compared to the GFP-MSC group. IL4 expression levels on day 3 in the IL4-MSC and IL4-pMSC groups were 77.7 ± 4.4 ng/mL and 34.6 ± 5.7 ng/mL, respectively. We have previously reported in vitro experiments in which the concentration of IL4 necessary for macrophage polarization and immunomodulation was greater than 20 ng/mL [41]. It is important to note that an initial transient acute inflammatory phase is a critical event for bone healing to occur [42], and continuous crosstalk between MSCs and macrophages is essential for the optimization of the healing process [43]. We implanted IL-4 MSCs within μRB scaffolds on the third day after the initial surgical defect was created so as not to interfere with the acute inflammatory phase, which lasts about 3 days and is essential for fracture healing in mice [44]; this resulted in enhanced bone formation in a murine critical-size bone defect model [45]. There are also several reports that local delivery of IL4 acutely inhibits osteogenic differentiation of MSCs [18,46]. In addition, we have reported that the injection of IL4-MSCs significantly decreased the number of empty lacunae compared to MSCs in a model of corticosteroid induced osteonecrosis but resulted in less osteogenesis in vivo [20]. This negative anti-inflammatory effect of IL4 on osteogenic differentiation may have led to the decreased osteogenesis of IL4-MSCs and IL4-pMSCs in this study.

PDGF-BB stimulates MSCs, promoting angiogenesis and osteogenesis. PDGF-BB secreted by preosteoclasts may determine the spatial and temporal vascularization required for support of osteogenesis [21]. We investigated the efficacy of CD in combination with MSCs and PDGF-BB-MSCs with injectable hydrogels as a cell-based therapy for the treatment of steroid-associated ONFH in rabbits. The injection of PDGF-BB-MSCs significantly decreased the number of empty lacunae and increased angiogenesis compared to MSCs. In addition, CD with an injection of PDGF-BB-MSCs had a trend towards greater osteogenic differentiation than the CD alone group [47]. Regarding osteogenic differentiation in our in vitro studies, the PDGF-BB-MSC group showed the best results among all eight groups. The percentage of Alizarin red-stained area in the PDGF-BB-MSC group was higher than the IL4-PDGF-BB-pMSC. On the other hand, there was no difference in the percentage of ALP and Alizarin red-stained area between GFP-MSC and IL4-PDGF-BB-pMSC groups. In addition, the percentage of Alizarin red-stained area in the IL4-PDGF-BB-pMSC group was higher than the IL4-PDGF-BB-MSC group (Figure 6). PDGF-BB expression levels on day 3 in the PDGF-BB-MSC, IL4-PDGF-BB-MSC, and IL4-PDGF-BB-pMSC groups were 121.7 ± 20.1 pg/mL, 8.7 ± 2.0 pg/mL, and 71.5 ± 7.8 pg/mL, respectively. These results suggest that the outcome of osteogenic differentiation was dependent on the PDGF-BB expression level.

The IL4 expression level in the IL4-MSC group and the PDGF-BB expression level in the PDGF-BB-MSC group was higher than in the IL4-pMSC and PDGF-BB-pMSC groups, respectively. These phenomena may be attributed to the inferior cell proliferative ability of IL4-pMSCs and PDGF-BB-pMSCs compared to IL4-MSCs and PDGF-BB-MSCs, respectively. On the other hand, the IL4 and PDGF-BB expression levels in the IL4-PDGF-BB-pMSC group were higher than the IL4-PDGF-BB-MSC group. This phenomenon may be due to some synergistic effect of preconditioning MSCs with co-over-expression of IL4 and PDGF-BB, but this is a subject for future research.

There is one other report on the co-over-expression of genetically modified MSCs with two different agents using the lentiviral vector. The added genes were vascular endothelial growth factor and B-cell lymphoma-2; the co-over-expressing MSCs showed significantly higher expression of each agent than singly modified MSC [48]. However, in the present study, the expression of each substance was lower in MSCs co-over-expressing IL4 and PDGF-BB than in MSCs recombinantly expressing each single gene. This phenomenon may be due to the combination of agents to be co-over-expressed. Preconditioning IL4-PDGF-BB-MSCs, in which IL4 and PDGF-BB were co-over-expressed, increased the expression of both IL4 and PDGF-BB in this study. The reason for this phenomenon is unknown, but the stimulatory effect of preconditioning on overall cell function is well documented. It is worth noting that in the IL4-PDGF-BB-pMSC group, the IL4 expression level on day 3 was greater than 20 ng/mL and thus was potentially sufficient to exert a biological effect of IL4 [41]. The percentage of ALP and Alizarin red-stained area of IL4-PDGF-BB-pMSC group was superior to that of the GFP-MSC group. These results indicate that the potential adverse effects of IL4 on early inflammation and bone formation might be overcome by the PDGF-BB released from the IL4-PDGF-BB-pMSCs. The IL4-PDGF-BB-pMSCs will potentially increase angiogenesis and osteogenesis and decrease the number of empty lacunae in vivo, properties important to the early treatment for ONFH.

## 5. Conclusions

This in vitro study investigated the efficacy of genetically modified MSCs (IL4-MSCs, PDGF-BB-MSCs, and IL4-PDGF-BB-MSCs) and corresponding preconditioning of these MSCs as a potential adjunctive cell-based therapy for CD. The use of PDGF-BB-MSCs or IL4-PDGF-BB-pMSCs increased cell proliferation compared to MSCs alone. Furthermore, PDGF-BB-MSCs promoted osteogenic differentiation compared to MSCs alone. Although osteogenic differentiation for MSCs alone and IL4-PDGF-BB-pMSCs showed no difference, IL4-PDGF-BB-pMSCs co-expressed sufficient amounts to exert a biological effect IL4 and PDGF-BB and could be expected to have significant osteogenic differentiation potential in vivo. Thus, these genetically modified MSCs may provide a useful supplementary cell-based therapy for CD.

## Figures and Tables

**Table 1:** Fold change in the amount of dsDNA compared to day 1.

	Day 3	Day 5	Day 7
GFP-MSCs	1.8 ± 0.1	4.4 ± 0.1	5.8 ± 0.1
GFP-pMSCs	2.2 ± 0.2	4.0 ± 0.3	5.5 ± 0.5
IL4-MSCs	6.8 ± 0.5	20.2 ± 2.4	30.4 ± 4.4
IL4-pMSCs	7.3 ± 0.1	12.5 ± 0.6	19.9 ± 3.0
PDGF-BB-MSCs	3.3 ± 0.7	12.5 ± 0.6	27.4 ± 1.7
PDGF-BB-pMSCs	2.3 ± 0.1	6.7 ± 0.1	12.6 ± 0.2
IL4-PDGF-BB-MSCs	6.4 ± 1.3	20.5 ± 4.2	26.7 ± 3.7
IL4-PDGF-BB-pMSCs	5.5 ± 1.1	11.1 ± 1.7	16.3 ± 2.9

Abbreviations: MSCs, mesenchymal stromal cells; pMSCs, preconditioning MSCs.

**Table 2:** The summary of the results.

	GFP-MSCs	GFP-pMSCs	IL4-MSCs	IL4-pMSCs	PDGF-BB-MSCs	PDGF-BB-pMSCs	IL4-PDGF-BB-MSCs	IL4-PDGF-BB-pMSCs
Cell proliferation	+	+	+++	++	+++	++	+++	++
IL4/PDGF-BB expression	−/−	−/−	+++/−	++/−	−/+++	−/++	+/+	++/++
Osteogenic differentiation	++	++	−	−	+++	++	+	++

−, negative; +, positive; ++, strong positive; +++, very strong positive.

## Data Availability

All data are included in the paper.
